# A WUSCHEL-related homeobox 3B gene, *depilous (dep)*, confers glabrousness of rice leaves and glumes

**DOI:** 10.1186/1939-8433-5-28

**Published:** 2012-10-02

**Authors:** Rosalyn B Angeles-Shim, Kenji Asano, Tomonori Takashi, Junghyun Shim, Takeshi Kuroha, Madoka Ayano, Motoyuki Ashikari

**Affiliations:** 1 Bioscience and Biotechnology Center, Nagoya University, Chikusa, Nagoya, Aichi, 464-8601 Japan; 2 Honda Research Institute Japan, Kazusa-Kamatari, Kisarazu-shi, Chiba, 292-0818 Japan; 3 Plant Breeding, Genetics and Biotechnology Division, International Rice Research Institute, DAPO Box 7777, Metro Manila, Philippines; 4 Upland Farming Research Division, NARO Hokkaido Agricultural Research Center, 9-4 Shinsei-minami, Memuro, Kasai, Hokkaido 082-0081 Japan

**Keywords:** *depilous*, Rice, Trichomes, Glabrous, Leaves, Glumes, WUSCHEL-related homeobox

## Abstract

**Background:**

Glabrousness is an important agricultural trait for the practical breeding of rice. In this study, *depilous (dep),* the gene responsible for glabrous leaves and glumes of rice was identified by map-based cloning.

**Results:**

The *dep* gene encodes a WUSCHEL-related homeobox 3B that was fine-mapped to a 22-kb region on the short arm of chromosome 5 using progenies derived from crosses between Koshihikari (pubescent) and GLSL15, an *Oryza glaberrima* chromosome segment substitution line (glabrous). Complementation tests confirmed the conditioning of the glabrous phenotype by the *dep* gene. Phylogenetic analysis showed that *dep* groups with the WOX3 family of plant-specific homeobox transcription factors that are involved in regulating lateral organ development. Localization of *dep* in the nucleus indicates the function of the gene as a transcription factor. Spatial expression of the gene was observed in the base of young shoots, the leaf sheath, midrib, young roots and nodal structures.

**Conclusion:**

The identification and cloning of *dep* will not only provide basis for future research on the elucidation of the molecular mechanisms underlying trichome formation in rice but will also aid in breeding programs for the development of glabrous varieties.

**Electronic supplementary material:**

The online version of this article (doi:10.1186/1939-8433-5-28) contains supplementary material, which is available to authorized users.

## Background

Trichomes are specialized structures that originate from the aerial epidermis and develop as hair-like projections that extend from the epidermal surface ([Serna and Martin 2006];[Werker 2000];[Johnson 1975]). They differ considerably in cellular composition, density, morphology, location and, depending on whether they are glandular or non-glandular, in function. In most plant species, non-glandular trichomes function to lessen the heat load of leaves, reduce transpiration rates, and enhance freezing tolerance and herbivore resistance. Glandular trichomes on the other hand, not only offer physical resistance but also provide chemical protection against biotic and abiotic challenges ([Serna and Martin 2006];[Martin and Glover 2007]; Bandyopadhyay et al.[2004];[Werker 2000];[Mauricio and Rauscher 1997];[Johnson 1975]).

In rice (O*ryza sativa*), trichomes on the leaf blade take the form of hairs or bristles (Yamamoto et al.[1997];[Hoshikawa 1975]). The hairs are two-celled, cylindrical appendages that are arranged between nerves on the epidermis on either side of the leaves. Bristle type trichomes on the other hand, are one- or two-celled outgrowths, and may be of type Ia, Ib or II. The bristle type Ia trichomes are commonly found on the adaxial side of the leaf and are slightly longer than the type Ib. Both the hair and the bristle type II trichomes are located on the adaxial and abaxial leaf surfaces. The bristle type Ia and Ib trichomes as well as the hairs run perpendicular to the leaf surface, whereas the bristle type II are oriented parallel to the leaf blade. Both bristle type Ia and Ib trichomes are conical in shape despite differences in size and location, indicating that they may have been derived from the same origin (Yamamoto et al.[1997]). The bristle type trichomes, regardless of length or orientation, have also been described as prickle hairs (Islam et al.[2009]) or macrohair, whereas the hair has also been referred to as microhair (Kobayashi et al.[1997]; Shimizu et al.[1976]).

The reduction or absence of the bristle type trichomes on the surface of the leaves and the glumes defines the glabrous phenotype in rice. This trait has been considered as selectively neutral, with neither a known selective advantage nor disadvantage. However, in areas where rice culture remains manual, the glabrous trait is fairly valued since the lack of trichomes reduces skin irritation and itching among workers during harvesting, threshing, drying and milling of rice ([Rutger and Mackill 2001]; Makenzie et al.[1987]). In the U.S., cultivation of glabrous varieties has been an important part of the rice industry. Initially valued for hand harvesting and threshing because they cause less itching, the glabrous varieties became more prized due to their higher bulk density compared with pubescent varieties ([Rutger and Mackill 2001]). The higher bulk density due to the close packing of the glabrous kernels saves valuable space during storage and transportation (Varnamkhasti et al.[2008];[Rutger and Mackill 2001]). Glabrous varieties have also been known to create less dust during processing. At present, virtually all U.S. rice varieties, as well as many Australian varieties that share U.S. parentage, are glabrous ([Rutger and Mackill 2001]).

The recessive allele *gl1* and its duplicate pair *gl2* have been reported to control glabrousness in both the leaves and glumes of rice (Yamamoto et al.[1997];[Nagao and Takahashi 1963];[Sastry and Seetharaman 1980]; Sato et al.[1990]). Linkage maps associated *gl1* with restriction fragment length polymorphism markers mapped on chromosome 5 (14.3±7.4 cM from RG182 and 20.9±8.3 cM from RG403) (Yu et al.[1992]; Yu et al.[1995]). To date, however, the gene controlling the trait has yet to be identified and characterized. We report here the identification and cloning of *depilous (dep),* the gene regulating trichome formation in rice leaves and glumes. This gene was identified by positional cloning using a previously identified glabrous chromosome segment substitution line (GLSL15) carrying an introgressed fragment from the African cultivated rice, *O. glaberrima* in the background of *O. sativa* subsp. *japonica* cv. Koshihikari. *dep* encodes a WUSCHEL-related homeobox 3B protein that belongs to a class of transcription factors regulating lateral organ development.

## Results

### Phenotype of the substitution line, GLSL15

**Figure 1:**
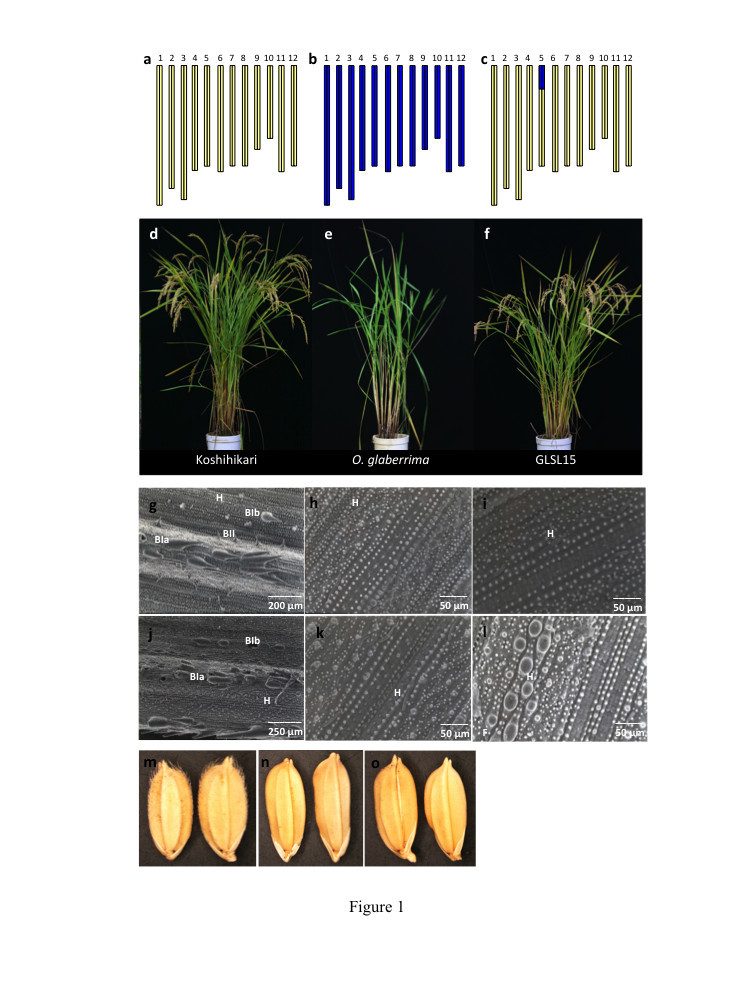
**Morphology and graphical genotypes of the plant materials** **.** Graphical representations of the genomic constitution of (**a**) Koshihikari, (**b**) *O. glaberrima* and (**c**) GLSL15. Gross morphology and overall structural variation among (**d**) Koshihikari (**e**) *O. glaberrima* (**f**) and GLSL15 during the reproductive stage. Scanning electron micrographs of the adaxial and abaxial leaf surfaces of (**g**, **j**) Koshihikari, (**h**, **k**) *O. glaberrima* and (**i**, **l**) GLSL15. (**m**) Koshihikari showing trichomes in the glumes, and (**n**) *O. glaberrima* and (**o**) GLSL15 exhibiting glabrousness or absence of trichomes on the glumes. H=hair, BIa=bristle type Ia, BIb-bristle type Ib, BII=bristle type II.

The substitution line GLSL15 shares the genomic constitution of cv. Koshihikari except for an approximately 3.15-Mb fragment of *O. glaberrima* on the short arm of chromosome 5 (Figure1a-c). Gross morphological examination showed that GLSL15 closely resembles Koshihikari (Figure1d-f) although scanning electron micrographs revealed that the substitution line is more similar to *O. glaberrima* in leaf ultrastructure (Figure1g-l). Both GLSL15 and *O. glaberrima* lack the bristle-type trichomes and have hairs only on both the abaxial and adaxial surface of the leaves. The hairs run parallel to the leaf venation and are located beside the motor cells or stomata. Koshihikari leaves, on the other hand, have both hair and the bristle-type trichomes. Bristle type Ia, Ib and II trichomes were observed on the adaxial leaf surface, whereas only bristle type Ia and Ib were seen on the abaxial leaf surface. The bristle-type trichomes are located on silica cells over a thin vascular bundle and are oriented perpendicular or parallel to the leaf blade. Trichomes that are prominent on the glumes of Koshihikari are absent on the glumes of both GLSL15 and *O. glaberrima* (Figure1m-o)*.*

### Genetic analysis and fine-scale mapping of the *dep* gene

**Figure 2:**
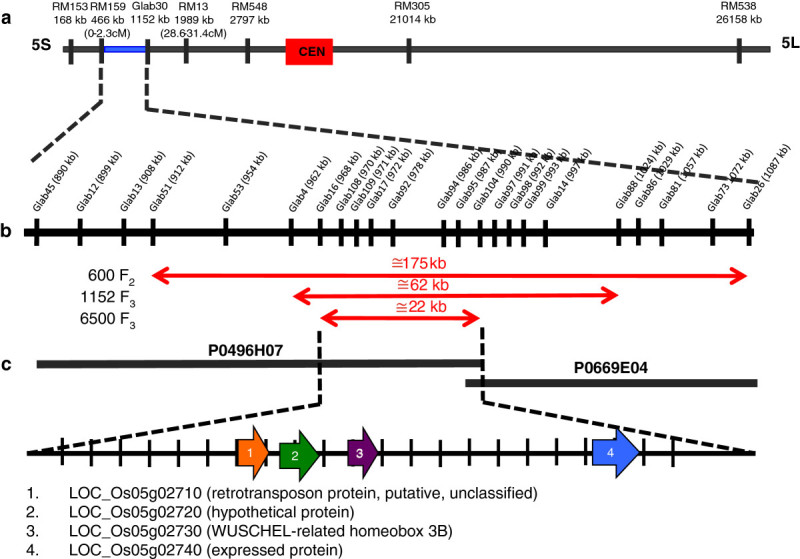
**Linkage analysis and fine-scale mapping of** ***dep.*** (**a**) Mapping of the *dep* gene on the short arm of chromosome 5. The blue bar represents the putative location of the gene. (**b**) Fine mapping of *dep* using F_2_ lines generated from crosses between Koshihikari and GLSL15, and F_3_ lines derived from F_2_ lines with informative recombination points. Red, double-headed arrows show the candidate region identified for each of the populations genotyped. (**c**) Annotated genes covered within the BAC clones spanning the 22-kb candidate region. Figure not drawn to scale.

Linkage analysis using 600 F_2_ lines generated from crosses between Koshihikari and GLSL15 showed that the gene controlling glabrousness in rice, herein named *depilous (dep)*, is tightly linked to SSR markers RM159 (0–2.3 cM) and RM13 (28.6-31.4 cM) on the short arm of chromosome 5 (Figure2a). Fine mapping of the *dep* gene using the same 600 F_2_ lines and newly developed indel-based and dCAPs markers narrowed down the candidate region to 175 kb. This region was further delimited to 62 kb by fine mapping using an initial set of 1152 F_3_ plants generated from the F_2_ lines with informative recombination points. Genotyping of an additional 6500 F_3_ lines finally identified a 22-kb candidate region for the gene (Figure2b). This 22-kb stretch is covered in a single BAC (P0496H07) and includes 4 annotated genes encoding the ORF for a retrotransposon protein (LOC_Os5g02710), a hypothetical protein (LOC_Os05g02720), a WUSCHEL-related homeobox 3B (LOC_Os05g2730) and an expressed protein (LOC_Os05g2740) (Figure2c). The observed Mendelian ratio for pubescent and glabrous types in the F_2_ population indicates a monogenic control and recessive inheritance of the trait although a slight segregation distortion was observed (data not shown).

### Map-based cloning of the *dep* gene

**Figure 3:**
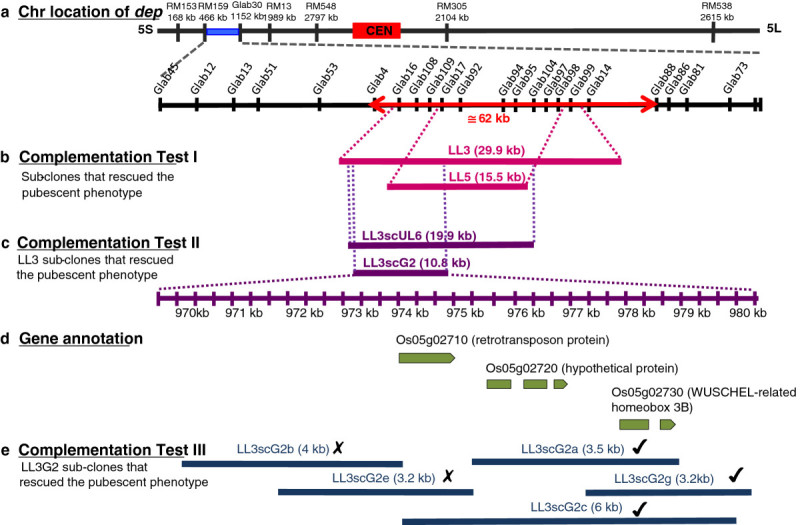
**Map-based cloning of** ***dep.*** (**a**) Rough and fine map basis of complementation tests carried out to identify the gene responsible for the glabrous phenotype in rice. Red-, double-headed arrow indicates the 62-kb candidate region identified for *dep*. (**b**) BAC sub-clones used in the initial complementation test that restored the pubescence of the substitution line used for transformation. (**c**) Sub-clones generated from the LL3 fragment that complemented the glabrous phenotype in the substitution line used for transformation. (**d**) Annotated genes covered within the LL3scG2 sub-clone. (**e**) Sub-clones derived from LL3scG2 fragment that were used to transform a glabrous substitution line of rice. Lines that are marked with a cross failed to complement the glabrous phenotype whereas those marked with a check restored the pubescence in T_0_ plants. Figure not drawn to scale.

**Figure 4:**
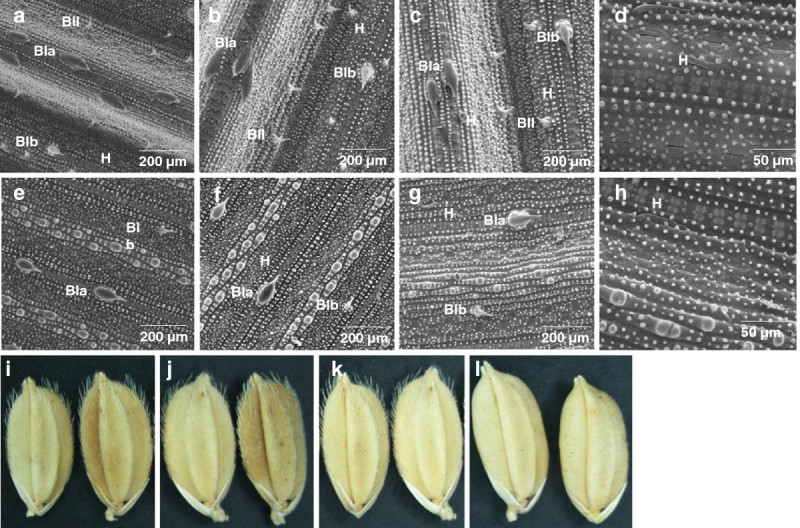
**Restoration of the pubescent phenotype in a glabrous substitution line (cv. T65 background).** Scanning electron micrographs of the adaxial and abaxial leaf surfaces of transgenic plants (T_0_) carrying the LL3scG2a (**a**, **e**), LL3scG2c (**b**, **f**) and LL3scG2g (**c**, **g**) genomic fragments, exhibiting recovery of bristle type trichomes. Hairy glumes of transgenic plants (T_0_) carrying the (**i**) LL3scG2a, (**j**) LL3scG2c and (**k**) LL3scG2g genomic fragments. Scanning electron micrographs of the leaves of the vector control showing absence of trichomes on both the (**d**) adaxial and (**h**) abaxial sides of the leaf, as well as on the (**l**) glumes. H=hair, BIa=bristle type Ia, BIb-bristle type Ib, BII=bristle type II.

To determine which of the candidate genes regulate trichome formation in rice, a series of complementation tests were carried out parallel to the fine-scale mapping. Six overlapping fragments covering the initially identified 62 kb candidate region (Figure3a) were picked from a newly developed sub-library of a Koshihikari BAC clone. From these, 2 overlapping fragments spanning 30-kb (LL3) and 15-kb (LL5) restored the pubescent phenotype in a glabrous substitution line that was used for transformation (Figure3b). T_0_ plants carrying the LL3 or LL5 fragment showed bristle-type trichomes on both adaxial and abaxial leaf surfaces and also on the glumes of the spikelets. T_1_ plants of both LL3 and LL5 also had the same pubescent phenotype. Using the LL3 sub-clone, 5 overlapping fragments were generated and used for another round of complementation tests. From the 5 fragments, sub-clones LL3scUL6 and LL3scG2, spanning 20 and 11kb, respectively, recovered the pubescent phenotype of the glabrous substitution line used (Figure3c). T_0_ plants carrying the LL3scUL6 or LL3scG2 fragments had the bristle type trichomes on both the adaxial and abaxial leaf surface, as well as on the glumes. The LL3scG2 genomic fragment included the annotated open reading frames (ORFs) for LOC_Os05g02710, LOC_Os05g2720 and LOC_Os05g2730 (Figure3d). A third and final complementation test used the LL3scG2 sub-clone to generate 5 overlapping fragments i.e. LL3scG2a, LL3scG2b, LL3scG2e, LL3scG2g and LL3scG2c for transformation (Figure3e). From these, only LL3scG2a, LL3scG2c and L3scG2g spanning 3.5, 6 and 3.2 kb, respectively, restored the pubescent phenotype of the substitution line used for the complementation analysis. Transgenic plants carrying any of these genomic fragments showed bristle type trichomes on both adaxial and abaxial leaf surfaces, as well as on the glumes (Figure4). LL3scG2c covered the ORFs for the three candidate genes, whereas LL3scG2a included only the ORFs for LOC_Os05g2720 and LOC_Os05g2730. The LL3scG2g sub-clone covered only the ORF of LOC_Os05g2730 encoding a WUSCHEL-related homeobox 3B. These results indicate that LOC_Os05g2730 is the *dep* gene that conditions glabrousness in rice.

### Structure, phylogeny and comparative sequence analysis of *dep* encoding a WUSCHEL-related homeobox 3B protein

**Figure 5:**
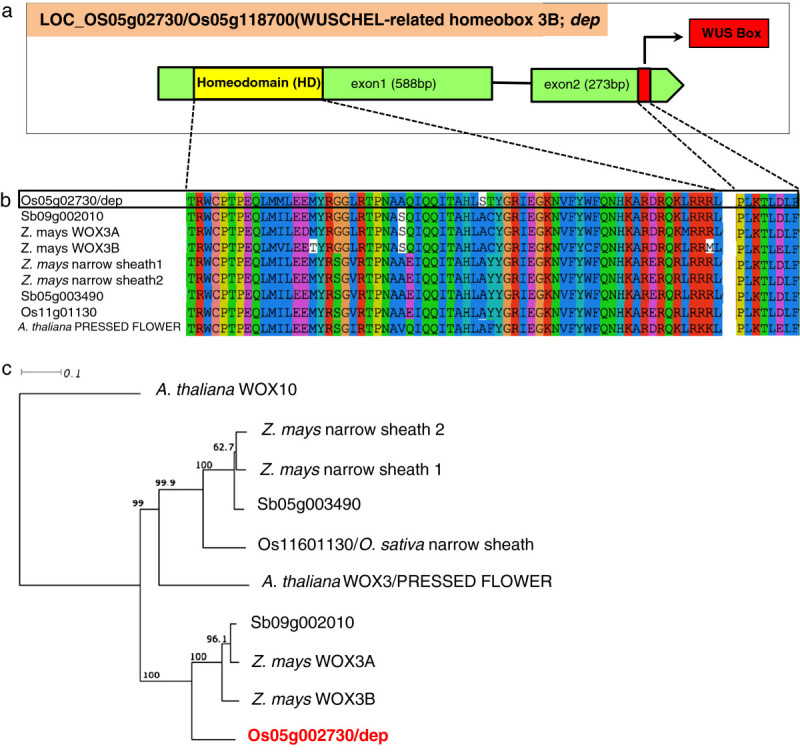
**Structure and phylogenetic relationship of** ***dep*** **with other WOX3 proteins.** (**a**) Schematic diagram of *dep*. (**b**) Amino acid alignment of the highly conserved homeodomain and WUS box of dep and WOX3 orthologues from *O. sativa, Z. mays*, *S. bicolor* and *A. thaliana*. (**c**) Phylogenetic analysis showing the relationship of dep with other members of the WOX3 family. *A. thaliana* WOX10 was used as an outgroup. Bootstrap percentages are shown in the branching points.

Combined fine mapping and complementation tests demonstrated that LOC_Os05g2730 corresponding to the *dep* gene regulates the glabrous phenotype in rice. The gene is 861 bp in length and encodes a WUSCHEL-related homeobox 3B protein composed of 286 amino acids (Figure5a). The dep protein is characterized by a homeobox domain in the N terminal and a WUS box motif in the C terminal. Amino acid sequences of both the homeobox domain and WUS box motif are highly conserved in *dep* and in the *WUSCHEL HOMEOBOX* (*WOX) 3* orthologues in *Arabidopsis thaliana,* maize *(Zea mays)* and sorghum (*Sorghum bicolor*) (Figure5b). Phylogenetic analysis using *A. thaliana WOX 10* as outgroup showed that *dep* groups with other *WOX3* relatives from *A. thaliana*, maize and sorghum, although it roots outside the *Arabidopsis WOX3/PRESSED FLOWER* (*PRS*)-maize *Narrow Sheath (NS)* branch (Figure5c).

Sequencing of *dep* showed 3 amino acid substitutions (G23V, V14A and T34A) and a serine deletion (S32del) in the *O. glaberrima* allele relative to the Koshihikari allele. To determine which of these mutations causes the glabrous phenotype, sequencing of *dep* in glabrous rice mutants, as well as in a natural population of pubescent and glabrous *O. glaberrima* lines was also carried out*.* Sequence analysis showed that both glabrous and pubescent lines of *O. glaberrima* carry the same amino acid substitutions and deletion relative to Koshihikari*.* In addition, *dep* sequence in the glabrous rice mutants studied was similar to that of Koshihikari. Together, these results indicate that none of the sequence variations observed is responsible for the glabrous trait in rice. Sequencing of the 2-kb region upstream of *dep* also did not show any difference between *O. glaberrima* and Koshihikari*.*

### Subcellular localization and spatial expression of *dep*

**Figure 6:**
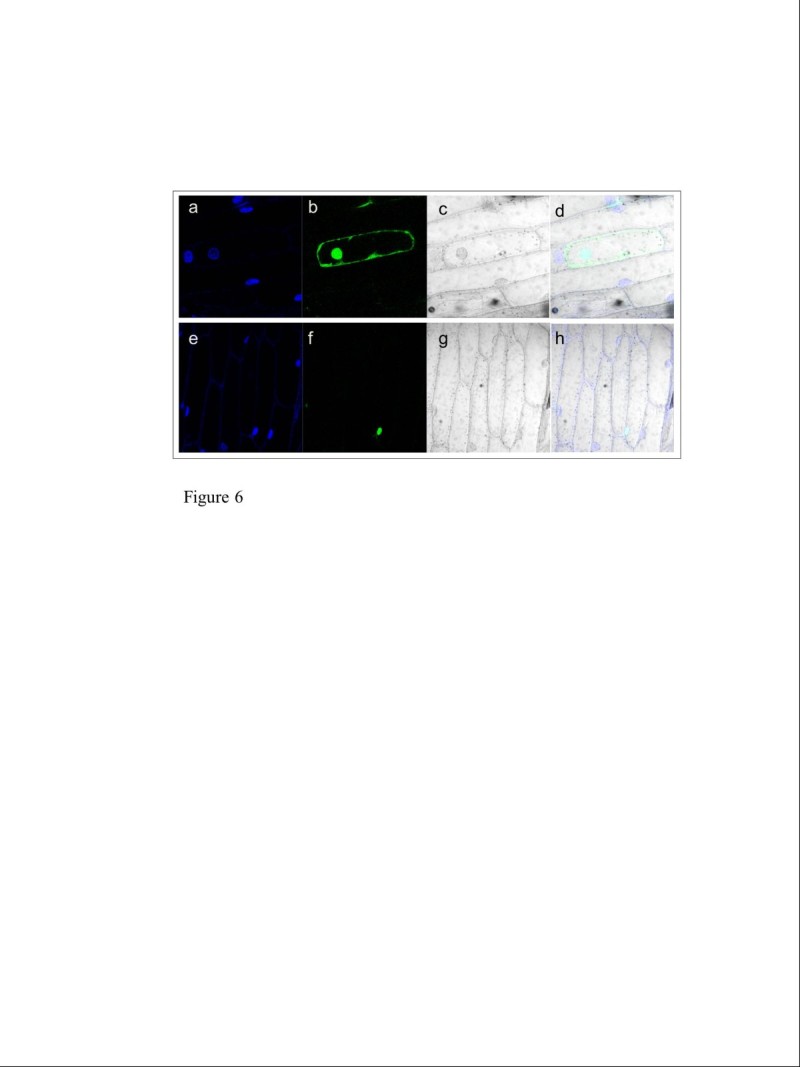
**Subcellular localization of the** ***dep-*** **GFP protein in onion epidermal cells analyzed by confocal laser microscopy.** DAPI stained, GFP, bright field and merged images of onion epidermal cells expressing 35S::*GFP* as a control (**a**-**d**) and *35S::dep-GFP* (**e**-**h**).

**Figure 7:**
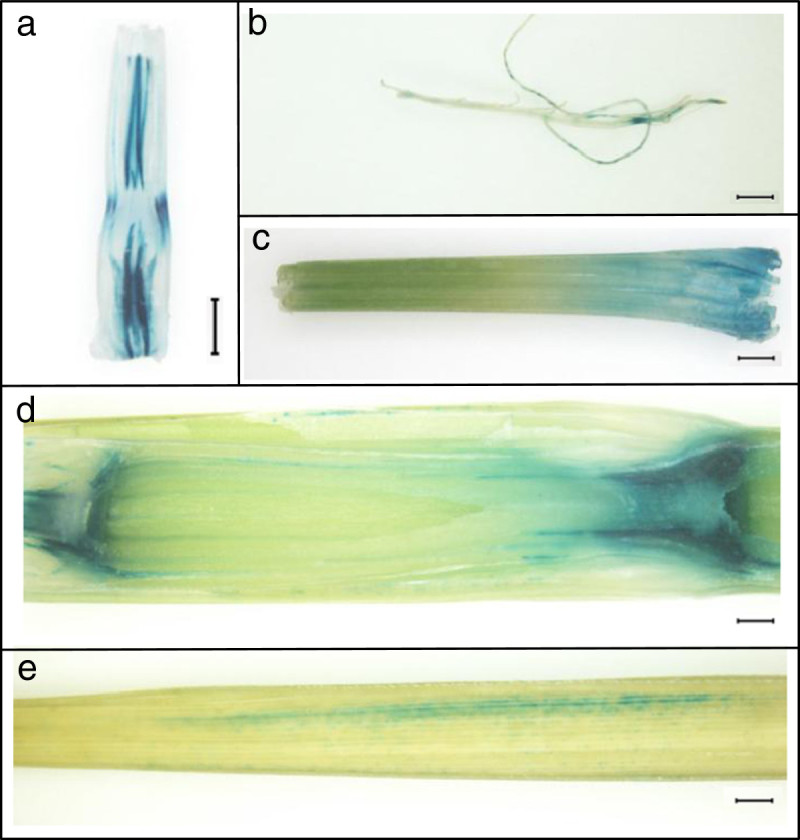
**Histochemical GUS assay using various organs of rice transformants carrying** ***dep*** **promoter-GUS.** GUS staining was observed in the (**a**) shoot base (longitudinal section), (**b**) lateral roots and (**c**) leaf sheath of 10-day-old plants as well as in the (**d**) nodal structures and (**e**) mid-vein of 20-day-old plants. Scale bar=1 mm.

Transient expression in onion epidermal cells of a translational fusion protein between *dep* and a GFP reporter gene showed that the gene localizes in the nucleus (Figure6). GUS activity in transgenic plants expressing the β-glucuronidase reporter gene fused to a *dep* promoter fragment was detected in the shoot base, lateral roots and leaf sheath of 10-day-old plants and in the nodes and mid-vein of 20-day-old plants (Figure7a-e).

## Discussion

Glabrous mutants exhibiting a few or total lack of trichomes in the leaves have been reported for a wide range of species. In *A. thaliana*, considerable progress has been made in elucidating the molecular mechanisms involved in trichome initiation, morphogenesis and patterning (Szymanski et al.[2000]). To date, the specification of trichome formation in *Arabidopsis* has been demonstrated to require the interaction of bHLH, MYB and WD repeat proteins in a functional complex (Zhao et al.[2008]; Tominaga et al.[2008]; Hauser et al.[2001]). In cotton (*Gossypium arboretum*), similar protein complexes have been reported to control trichome formation. *GaMYB2,* an *Arabidopsis GLABRA1* (*GL1)* orthologue in cotton, has been reported to have an amino acid motif for interaction with a bHLH protein. *GaMYB2* expressed in *A. thaliana* under the control of *GL1* promoter fully restored trichome formation in a *gl1* mutant. *GhMYB109* is another R2R3 MYB gene identified in upland cotton (*G. hirsutum*). Like *GaMYB2*, it also has a conserved motif for interaction with R-like bHLH proteins. Two other functional homologues of the *Arabidopsis TRANSPARENT TESTA GLABRA* were identified in cotton and both genes encode WD repeat proteins (Humphries et al.[2005]; Wang et al.[2004]; Suo et al.[2003];[Serna and Martin 2006]). Despite parallelisms in the model system for trichome regulation in *Arabidopsis* and cotton, similar multimeric complexes among MYB, bHLH and WD protein repeats have not been associated with trichome formation in species outside the Rosid division to which *Arabidopsis* and cotton both belong. In fact, different regulatory genes have been demonstrated to induce the formation of the various types of trichomes, as in the case of Asterids ([Serna and Martin 2006]). Furthermore, many plant species produce different types of trichomes that appear to be independently regulated (Moose et al.[2004]).

In our study, *dep*, the gene responsible for the glabrous phenotype in rice, was mapped and identified by positional cloning. The rice substitution line, GLSL15 exhibits a glabrous phenotype that is characterized by the absence of trichomes on the leaf blade and the glumes. This line has the full genomic constitution of cv. Koshihikari except for a 3.15-Mb stretch of chromosome 5 of *O. glaberrima.* Glabrousness in this substitution line has been associated with the introgressed fragment of *O. glaberrima* (Shim et al.[2010]).

Rough mapping of *dep* identified the location of the gene between 0–2.3 cM (RM159) and 28.6-31.4 cM (RM13) on the short arm of chromosome 5, coinciding with the reported rice *gl1* identified between 3.8 and 24.8 cM, also on the short arm of chromosome 5. Genotyping and phenotyping of a total of 8252 F_2_ and F_3_ lines showed a monogenic and recessive control of the trait similar to previous reports on the monogenic control of glabrousness (Li et al.[2010];[Foster and Rutger 1978]). A slight distortion in genetic segregation for the trait was observed and this may be due to the preferential fertilization of a particular gametic genotype. Segregation distortion has been frequently observed at the *gl1* locus in *indica* x glabrous *japonica* crosses and changes in the segregation ratio for *gl1* have been reportedly caused by the unequal chances of fertilization of the female gametes (Sato et al.[1990]).

Fine-scale mapping narrowed the candidate region for *dep* into a 22-kb stretch covering 4 annotated genes that encode ORFs for a retrotransposon protein (LOC_05g02710), a hypothetical protein (LOC_05g02720), a WUSCHEL-related homoebox 3B (LOC_05g02730, *dep*) and an expressed protein (LOC_05g02740). *In silico* analysis showed that LOC_05g02710 contains a transposase DDE domain that has not been previously associated with any regulatory pathways for trichome formation. Blastp searches showed that LOC_05g02720 has a MYB/SANT-like DNA binding domain that has been reported to play regulatory roles in defense response and developmental processes in plants (Barg et al.[2005]; Yanhui et al.[2006]). In *Arabidopsis*, small MYB proteins including TRIPTYCHON (TRY), CAPRICE (CPC), TRICHOMELESS1 and ENHANCER OF TRY AND CPC 1, 2 and 3 have been identified as negative regulators of trichome initiation and patterning in the shoots (Wada et al.[1997]; Kirik et al.[2004]; Wang et al.[2008]). However, such regulatory proteins have been associated with trichome formation in species only within the Rosid division ([Serna and Martin 2006]; Moose et al.[2004]). LOC_05g02740 is a single-copy gene with a predicted protein length of 56 amino acids. Blastp searches indicate that orthologues of this gene in maize and sorghum have not been characterized as well. Another gene located within the candidate region is LOC_05g02730, which encodes a WUSCHEL-related homeobox 3B. WUSCHEL homeobox genes belong to a family of plant-specific transcription factors that have been shown to carry out specialized functions in key developmental processes in plants, including stem cell fate regulation, embryonic patterning and organ formation (Van der Graaf et al.[2009]; Haecker et al.[2004]). Expression and functional analysis of *WOX3* in *Arabidopsis* (*PRS*) and maize (*NS1* and *NS2*) demonstrated that *WOX3* is required in the recruitment of founder cells from the lateral compartment of shoot meristems that will later form the lateral and marginal regions of leaves and leaf orthologues. Null mutations in *PRS* of *Arabidopsis* resulted in defects in lateral sepal development and the elimination of the knife-edge cells in the marginal regions of the abaxial and adaxial sepal. Overexpression of the gene, on the other hand, resulted in the formation of multicellular bulges with trichomes on the stem and peduncle of the plant. Such structures were shown to be outgrowths of the epidermal cells ([Matsumoto and Okada 2001]). In maize, *ns1* and *ns2* mutations resulted in narrow leaves, with complete deletion of tooth hairs on the margins, as well as stem curvature and shortened internodes (Scanlon et al.[1996]).

In our study, complementation tests showed that genomic fragments covering the ORF of *dep* could restore the formation of bristle type trichomes on the leaf blade and the glumes of glabrous rice, indicating the role of this gene in trichome formation. However, it does not influence the formation of aerial appendages in the leaf margin. Phylogenetic analysis showed that *dep* groups with the reported *WOX3* branch carrying duplications of the *PRS/NS* branch in the *WOX3* lineage. While members of both clades relate to leaf development, *PRS/NS* orthologues are expressed in the lateral domain whereas those of the *WOX3* duplicate clade are expressed in the base of developing phytomers in the shoot meristem (Shimizu et al.[2009]; Nardmann et al.[2007];[Nardmann and Werr 2006]). Such differences in expression domains have been shown to be indicative of sub-functionalization of duplicate genes as a means to escape mutational decay (Nardmann et al.[2007];[Lynch and Force 2000]). Grouping of *dep* with the members of the *WOX3* duplicate branch suggests that *dep* may have arisen as a duplicate gene and was preserved by acquiring a sub-function that differs with respect to tissue expression specificity. This may account for the ability of *dep* to affect trichome formation in the leaf blade and glumes but not in the margins of the leaves. The localization of *dep* in the nucleus is consistent with the reported features of transcription factors.

Comparative sequence analysis for *dep* failed to identify the causal mutation that conditions the glabrous phenotype in rice, suggesting a possible epigenetic regulation of the gene responsible for the trait.

## Conclusions

Despite being a selectively neutral trait, interest in the glabrous phenotype in rice remains due to the practical advantages of cultivating glabrous varieties. In this study, *dep* encoding a WUSCHEL-related homeobox 3B protein was identified as the gene controlling glabrousness in rice leaves and glumes. The identification and cloning of *dep* will not only provide basis for future research on the elucidation of the molecular mechanisms underlying trichome development (i.e. initiation, distribution and patterning) in rice, but will also facilitate the efficient breeding for glabrous rice varieties using marker-aided selection.

## Methods

### Plant materials

*Oryza glaberrima* Acc IRGC104038, *O. sativa* subsp *japonica* cv. Koshihikari and a chromosome segment substitution line (GLSL15) previously identified as having glabrous leaves and glumes were used in this study (Shim et al.[2010]). The plant materials were either grown in the greenhouses of the Laboratory of Plant Molecular Biosystem or under natural conditions in the research field of Nagoya University, Togo, Aichi, Japan. Seedlings of the plant materials were first raised in the greenhouse for 30 days before transplanting them in the field.

### Leaf ultrastructure of glabrous rice plants

The presence or absence of trichomes on the leaves of 1 ½- or 2-month-old plants was determined by running a forefinger along the length of the leaf blade starting from the tip and going to the base while holding the tip of the leaf. Glabrous leaves offered no resistance to this action due to the absence of bristle type trichomes. To closely examine the leaf ultrastructure of the plant materials, scanning electron microscopy was employed. Leaves of Koshihikari, *O. glaberrima* and GLSL15 were sampled in vials, fixed in FAA (50% ethanol, formalin and glacial acetic acid at 18:1:1 ratio) solution and dehydrated using a graded series of acetone washes (30, 50, 70, 80, 90, 95, 99 and 100%). The samples were further dehydrated by critical point drying for 1 h at 35°C and 1200 psi using liquid CO_2_ as the transitional fluid. Samples were then mounted in a specimen stub, ion-coated and examined under a scanning electron microscope (Hitachi S-2600N). The presence of trichomes on the glumes was determined by manual examination and by checking the hulls under a light microscope.

### Linkage analysis and mapping of *dep*

To fine-map *dep*, a total of 600 F_2_ plants generated by crossing GLSL15 with Koshihikari, as well as 7652 F_3_ plants generated from F_2_ lines with informative recombination points, were genotyped using SSRs and newly developed insertion/deletion (indel)-based and derived cleaved amplified polymorphic sequence (dCAPS; Neff et al.[2002]) markers targeting the putative location of the gene. Genomic DNA from the mapping population was extracted using the TPS method. SSRs, and indel and dCAPS markers were amplified using standard PCR protocols and run in 3% agarose gel with ethidium bromide.

Indel and dCAPS markers were designed based on the existing sequence alignment of Nipponbare and 93–11, as well as sequence alignments of the initial 62-kb candidate region for *dep* in Koshihikari and *O. glaberrima*. The alignment was generated using the GENETYX-MAC/ATSQ (ver. 15.0.5) software.

### Complementation tests

Three sets of complementation tests were carried out parallel to fine mapping to identify the gene controlling glabrousness in rice. Koshihikari BAC (CRC26L16) covering the putative location of the glabrous gene was screened to confirm the presence of markers within the candidate gene region. To develop a sub-library of the clone, the BAC was either completely digested with *Asc* II and *Bam* HI, or partially digested with *Hind* III to generate differently sized fragments. These fragments were run in 1% low-melting agarose, eluted using the GELase system (Epicentre Biotechnologies, Madison, WI, USA) and ligated to the binary vector, TAC7. The binary vector was also digested with the same set of enzymes and separately treated with cow intestine alkaline phosphatase (CIAP) to prevent self-ligation. Ligation was performed using the Takara DNA Ligation Kit LONG (Takara, Otsu, Japan), following the manufacturer’s specifications. The recombinant plasmid was then transformed to *Escherichia coli* strain DH10B by electroporation and plated in LB medium with kanamycin. Plasmid DNA extraction followed by digestion using the appropriate enzyme was carried out to verify the presence of an insert. Alongside this, direct plasmid sequencing using the primer pair R3: 5′-AAT TAG GCC CGG GCG GAT-3′ and L3: 5′-GGC CGC GGC CGG CCG TCG −3′ was carried out to estimate the size and determine the map position of the insert. Recombinant plasmids harbouring inserts that cover the candidate gene region were separately introduced to *Agrobacterium tumefaciens* strain EH105 by electroporation (Hood et al.[1986]) and used to transform a glabrous substitution line in cv. T65 background following the methods of Hiei et al. ([1994]). Transformants were selected in a medium containing 50 mg hygromycin/l. The hygromycin-resistant plants were transplanted in the soil and maintained under greenhouse conditions at an ambient temperature of 30°C and 16 h light: 8 h dark photoperiod. Sub-clones used for subsequent complementation tests were derived from clones that restored the pubescent phenotype in the glabrous rice donor used for transformation.

### Phylogenetic and sequence analysis for *dep*

The genomic sequence of *dep*, along with the 2-kb region upstream of the gene was amplified by PCR from the genomic DNA of *O. glaberrima*, Koshihikari, glabrous rice mutants in a T65 background and a natural population of glabrous and pubescent *O. glaberrima* lines. The obtained amplicons were purified using the Promega Wizard Genomic DNA Purification Kit, sequenced using the Applied Biosystems 3130xl Genetic Analyzer and analysed using Genetyx-MAC/ATSQ. Orthologues of the translated *dep* were retrieved by homology searches using Blastx in the National Center for Biotechnology Information and Rice Annotation Project databases. The retrieved protein sequences were aligned by Clustalx ver. 2.0 using the default settings (Larkin et al.[2007]). Phylogenetic analysis based on the Neighbor-Joining method was performed using SplitsTree4 ver. 4.11.3 ([Huson and Bryant 2006]). The accuracy of the inferred phylogenetic relationship was determined by bootstrapping using 1000 replicates.

### Subcellular localization

The coding sequence of *dep* was ligated into the pA7-GFP plasmid vector to construct a *dep*-GFP fusion protein driven by the cauliflower mosaic virus 35S promoter. The constructs were then introduced into onion epidermal cells by particle bombardment. The GFP signal was viewed by confocal laser microscopy.

### Promoter-GUS assay

To determine the spatial expression of *dep*, the promoter region (including an 80-bp proximal region) of the gene was digested with the appropriate enzymes and fused to the GUS coding sequence via ligation to the pBl101 plasmid vector. The promoter-GUS fusion construct was then digested from the pBl101 plasmid, cloned into Hm-12 and transformed to *E. coli* by electroporation. Recombinant plasmid DNA extracted from *E. coli* was used for transfection in *A. tumefaciens* that was in turn used for plant transformation. Tissue samples from transgenic plants were stained for 12 h under dark conditions with X-Gluc solution (5.7 mM 5-bromo-4-chloro-3-indolyl-β-D-glucuronide, 1.5 mM K_3_Fe(CN)_6_, 0.9% Triton-X 100) before viewing under a stereomicroscope.

## Authors’ original submitted files for images

Below are the links to the authors’ original submitted files for images. Authors’ original file for figure 1 Authors’ original file for figure 2 Authors’ original file for figure 3 Authors’ original file for figure 4 Authors’ original file for figure 5 Authors’ original file for figure 6 Authors’ original file for figure 7
