# Supplementary figures and images for: A WUSCHEL-related homeobox 3B gene, depilous (dep), confers glabrousness of rice leaves and glumes
Source: Rice (N Y). 2012 Oct 2;5:28. doi: 10.1186/1939-8433-5-28 (PMC5520829; doi:10.1186/1939-8433-5-28)

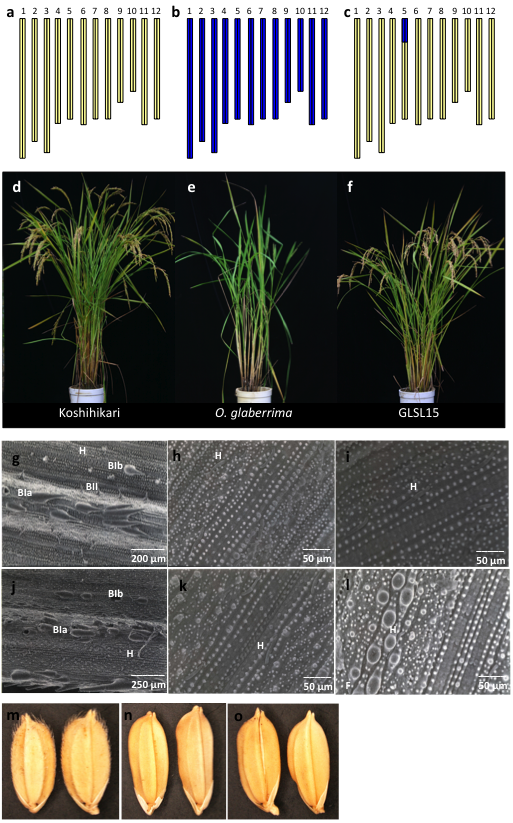

Supplement: Supplementary file 1 — Authors’ original file for figure 1 [file 12284_2011_19_MOESM1_ESM.tiff]

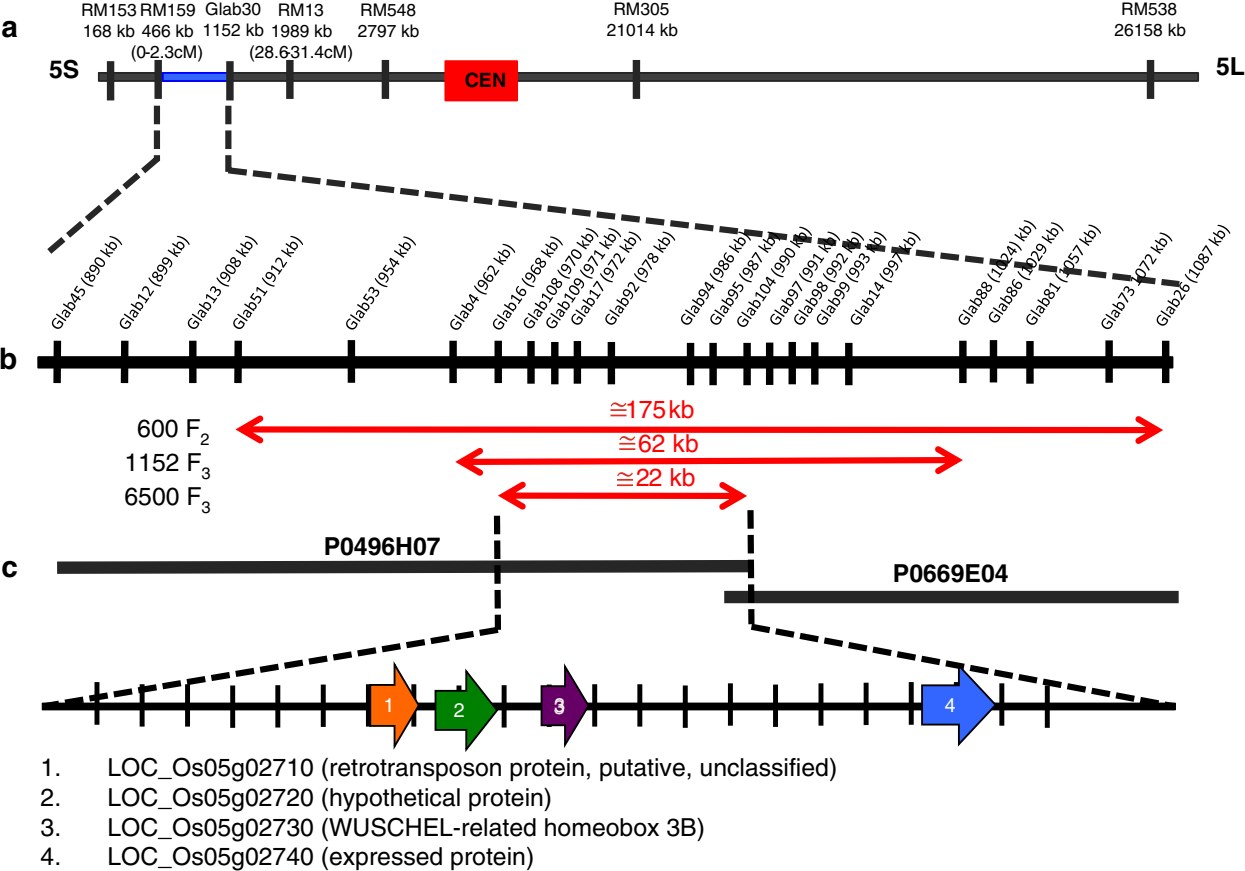

Supplement: Supplementary file 2 — Authors’ original file for figure 2 [file 12284_2011_19_MOESM2_ESM.pdf]

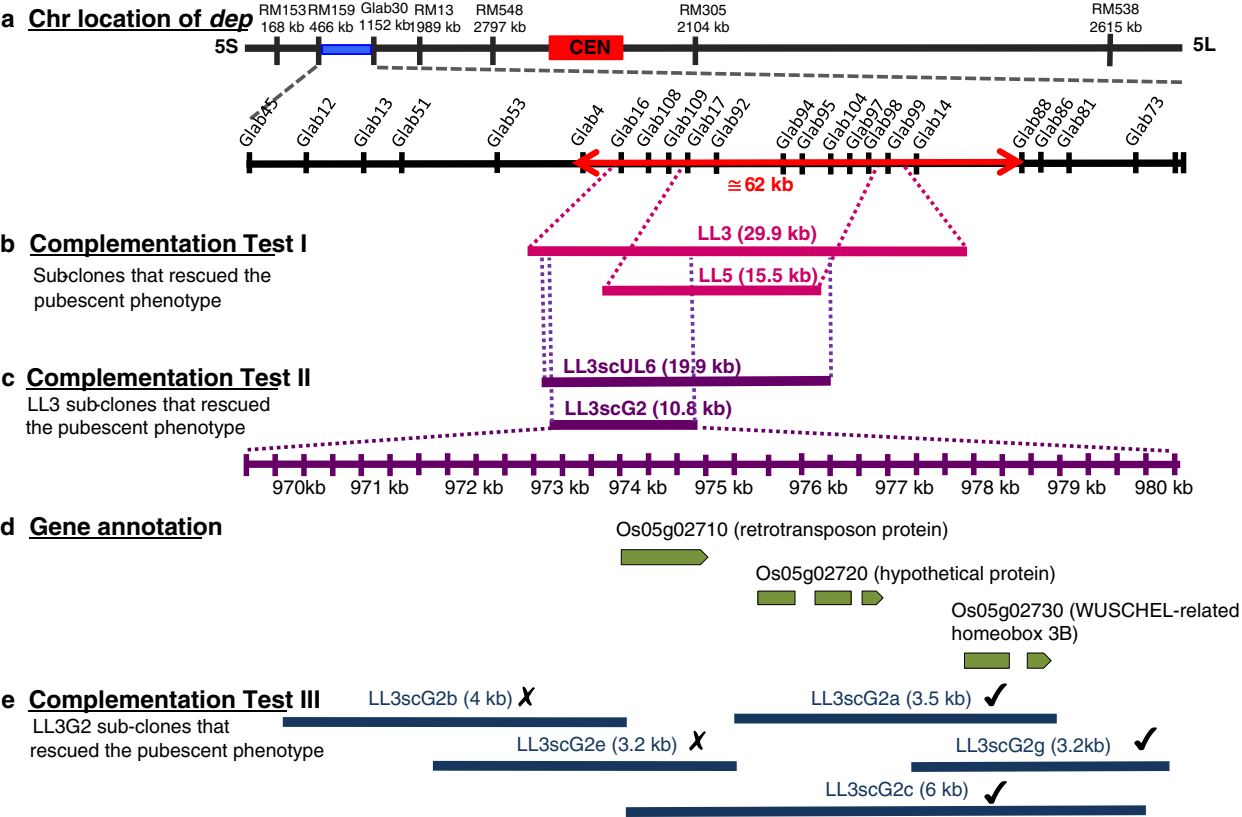

Supplement: Supplementary file 3 — Authors’ original file for figure 3 [file 12284_2011_19_MOESM3_ESM.pdf]

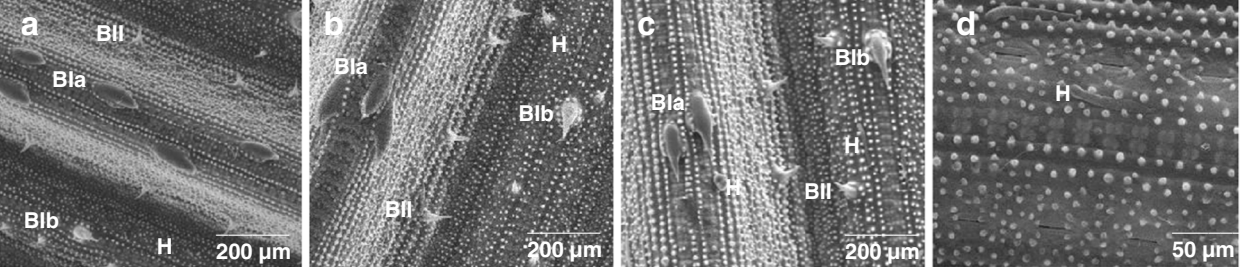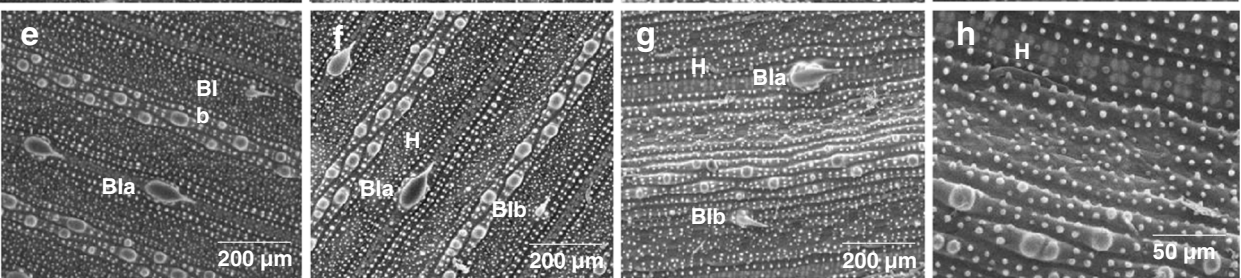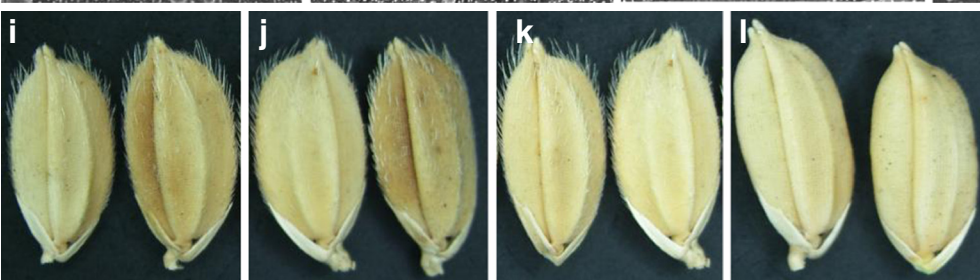

Supplement: Supplementary file 4 — Authors’ original file for figure 4 [file 12284_2011_19_MOESM4_ESM.pdf]

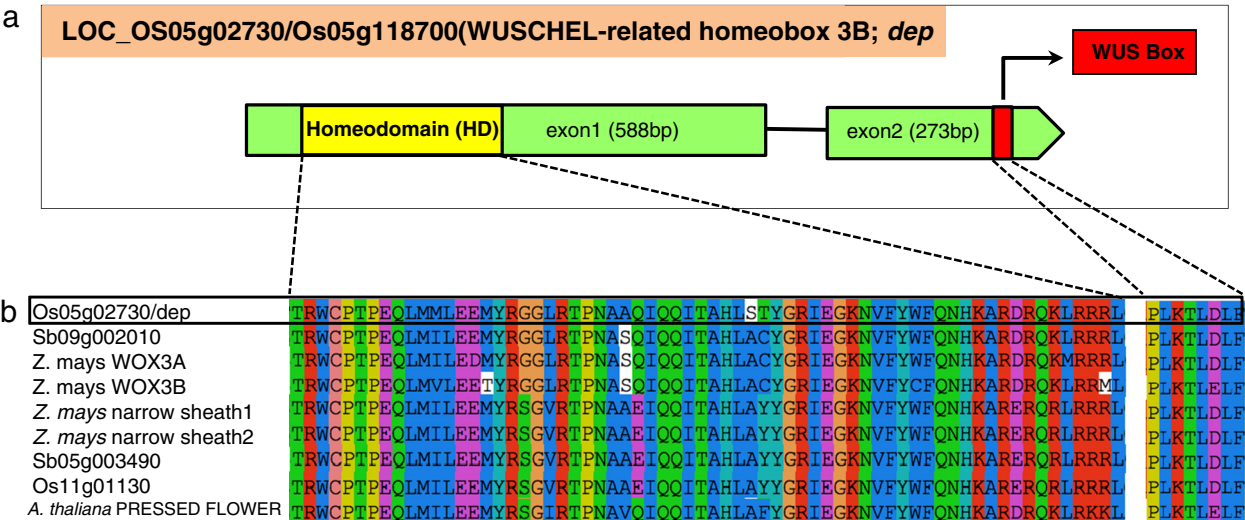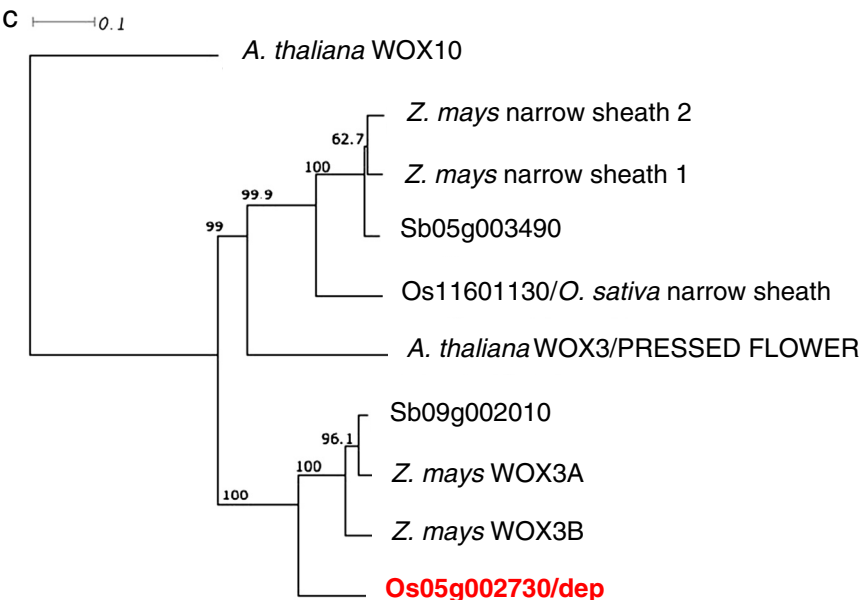

Supplement: Supplementary file 5 — Authors’ original file for figure 5 [file 12284_2011_19_MOESM5_ESM.pdf]

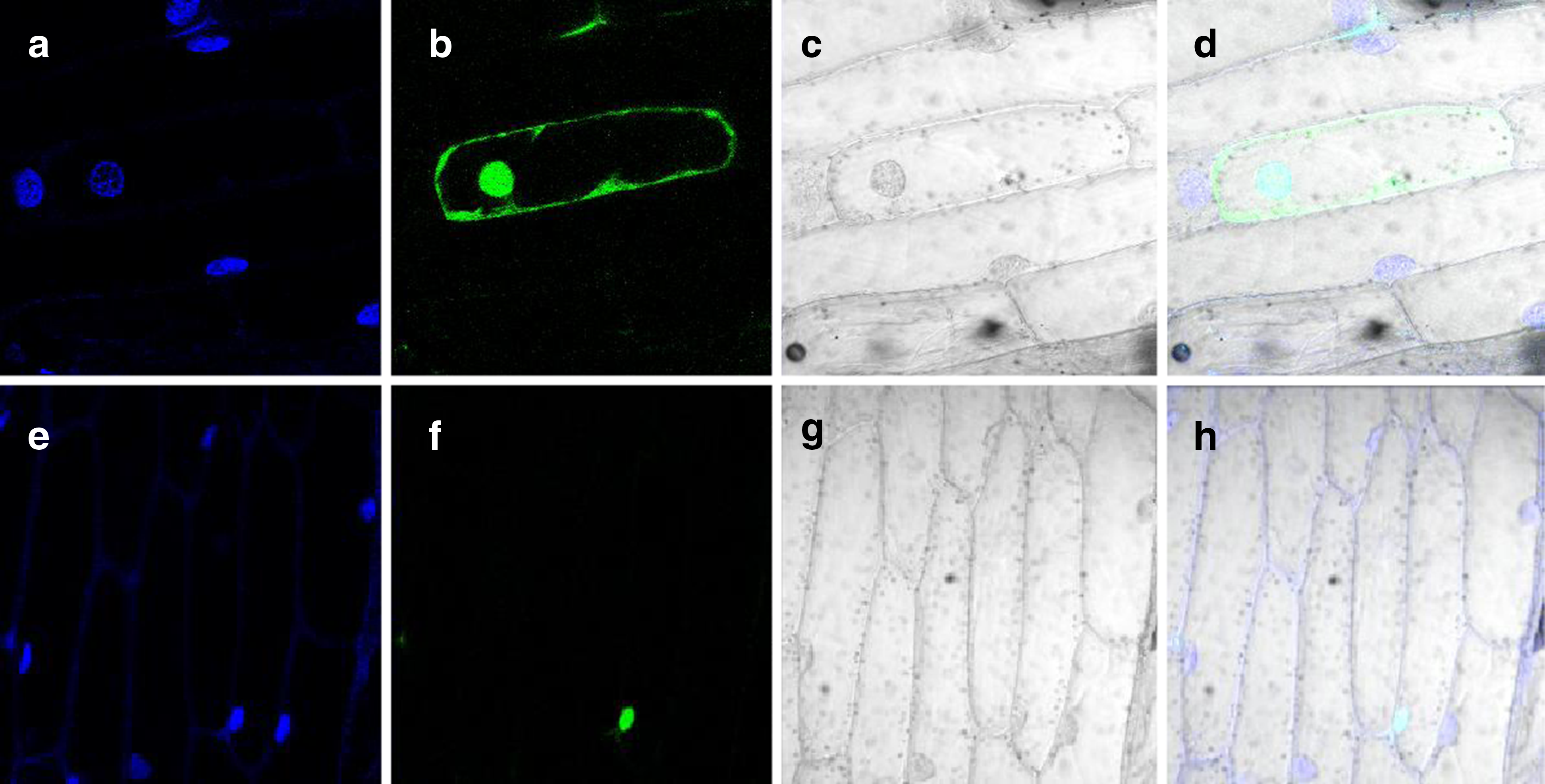

Supplement: Supplementary file 6 — Authors’ original file for figure 6 [file 12284_2011_19_MOESM6_ESM.tiff]

a

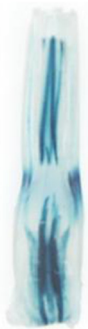

b

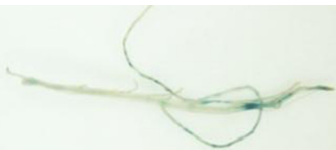

c

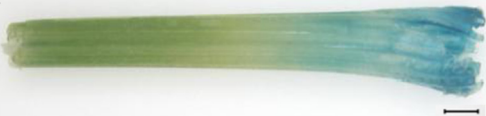

d

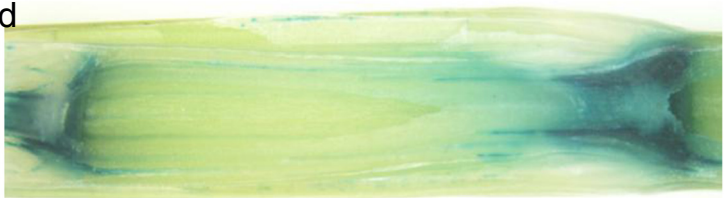

e

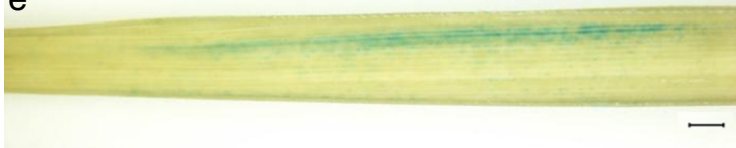

Supplement: Supplementary file 7 — Authors’ original file for figure 7 [file 12284_2011_19_MOESM7_ESM.pdf]
